# Differential photosynthetic responses to drought stress in peanut varieties: insights from transcriptome profiling and JIP-Test analysis

**DOI:** 10.1186/s12870-025-06984-y

**Published:** 2025-07-25

**Authors:** Jingyao Ren, Pei Guo, Xinhua Zhao, Xinlei Ma, Xin Ai, Jing Wang, Hongtao Zou, Haiqiu Yu

**Affiliations:** 1 College of Land and Environment, Shenyang Agricultural University, Shenyang, China; 2 College of Agronomy, Shenyang Agricultural University, Shenyang, China; 3 Liaoning Agriculture Vocational and Technical College, Yingkou, China

**Keywords:** Peanut, Drought stress, Photosynthesis, JIP-test, PSII performance

## Abstract

**Background:**

Drought stress poses a critical constraint to plant growth by impairing photosynthetic efficiency in crops.

**Results:**

Through transcriptome profiling of two peanut cultivars with contrasting drought tolerance, Fuhua18 (drought-sensitive, FH18) and Nonghua5 (drought-tolerant, NH5), we identified significant enrichment of differentially expressed genes in photosynthesis-related pathways. Notably, these genes were predominantly downregulated in FH18. Subsequent physiological analysis revealed cultivar-specific responses: Chlorophyll content decreased in FH18 but increased in NH5 after 24 h of drought treatment, accompanied by significant reductions in net photosynthetic rate (Pn) and water use efficiency (WUE) in both cultivars. The drought-induced physiological perturbations were further evidenced by elevated electrolyte leakage and activated antioxidant systems. To dissect photosynthetic apparatus dynamics, we implemented JIP-test analysis of chlorophyll fluorescence kinetics. Both cultivars exhibited substantial increases in Vj and Vi parameters at 24 h, while FH18 demonstrated a pronounced elevation in Vk during the O-J phase transition, suggesting severe impairment of the oxygen-evolving complex. Quantitative evaluation of photosynthetic performance indices revealed marked declines in PI_abs_ and PI_total_, indicating systemic damage to both PSI and PSII under drought stress. Comparative analysis identified 11 traits showing significant inter-cultivar variation, particularly in PSII reaction center parameters including PI_abs_, DI_0_/RC, RE_0_/RC, ABS/RC, and TR_0_/RC.

**Conclusion:**

These findings provide mechanistic insights into cultivar-dependent photosynthetic responses to drought stress, offering potential biomarkers for breeding drought-resilient peanut varieties.

**Supplementary Information:**

The online version contains supplementary material available at 10.1186/s12870-025-06984-y.

## Introduction

Plants are sensitive to abiotic stress, especially drought stress which has been one of the major adverse environmental stresses [1]. Water conditions and WUE affects the partitioning of plant assimilates, and significantly associated with crop yield under drought stress [2]. It has been found that in a drought-stressed environment, photosynthesis is one of the most sensitive metabolic processes, and that the responses of photosynthesis to drought stress are extremely complex [3]. It is generally accepted that there are two kinds of limitations on photosynthetic efficiency: stomatal and non-stomatal limitation [4]. In response to low or moderate drought stress, stomatal limitations are usually considered to be the dominant factor, while the photosynthesis organs in plants start to sustain damage as drought stress increases.

Drought stress induces multifaceted perturbations in photosynthetic machinery, with photoinhibition emerging as a critical limiting factor for carbon assimilation efficiency [5]. The imbalance between light energy absorption and utilization under water deficit conditions leads to over-excitation of photosystems, triggering a cascade of photodamage mechanisms. When stomatal closure restricts CO₂ availability and transpiration, excess excitation energy generates reactive oxygen species (ROS) through disrupted electron transport chain dynamics, creating a self-amplifying cycle of oxidative damage [6]. Current understanding identifies PSII as the primary vulnerability site, where drought-induced structural alterations in the oxygen-evolving complex (OEC) impair water-splitting function. In addition, PSI electron transfer capacity and redox state are reduced as a result [7]. The research on adzuki bean plants revealed that drought stress inhibited the physiological and biochemical characteristics, including the potential activity of photosystem II and photochemical efficiency at the early growth stage [8].

The fast chlorophyll a fluorescence kinetics (OJIP transient) has emerged as a powerful phenotyping tool for decoding PSII functionality under abiotic stress, enabling non-invasive tracking of PSII photoinactivation dynamics from initial electron transport imbalance to systemic photosystem collapse [9]. It has been introduced to explore the PSII antennae size, the reduction kinetics of the electron transport chain, the relative content of photosynthetic electron transport chain components, and damage to related protein functions [10, 11]. Modern OJIP analysis integrates quantum biophysics with machine learning algorithms to resolve three-dimensional excitation energy flows: (1) L-band (0.15 ms) quantifies light-harvesting complex II (LHCII) aggregation states, revealing early stomatal limitation-induced antenna overpacking, (2) electron transport through Q_A_-Q_B_ sites (K-band at 0.3 ms), detects oxygen-evolving complex (OEC) decoupling, and (3) plastoquinone pool redox dynamics (J-I phase at 2–30 ms), maps PSI acceptor-side limitation via plastocyanin redox kinetics [12]. Drought stress compromises electron transport dynamics at both donor (OEC impairment) and acceptor sides of PSII, disrupts energetic connectivity between PSII subunits, and concurrently diminishes PSI electron transfer capacity and redox state [7].

Peanut provides high-quality proteins and peanut byproducts, is an important economic crop cultivated worldwide. It grown in the arid and semi-arid regions, where drought stress seriously affects the productivity. Therefore, developing drought-tolerant cultivars is the best strategy for combating water deficits. In our preliminary studies, we observed significant differences in various aspects such as phenotypic characteristics, root activity, and osmotic adjustment between peanut varieties with varying drought tolerance under drought stress conditions. ​​However, ​​the divergence​​ in photosynthetic response traits under drought stress was particularly pronounced, and a substantial number of genes along the photosynthetic pathways were differentially expressed. ​​Thus​​, in the present study, a comparative analysis photosynthesis between contrast drought tolerant varieties were performed. It benefits not only understand the photosynthetic physiology of peanuts in response to drought stress, but also to the drought-tolerant varieties breeding.

## Materials and methods

### Planting materials, growth conditions, and treatments

In a prior investigation, NH5 (bred by Shenyang Agricultural University) and FH18 (bred by Aeolian Sand Research Institute of Liaoning Academy of Agricultural Sciences) were determined to be drought-tolerant and drought-sensitive varieties, respectively [13]. Evenly sized seeds were sterilized and soaked in distilled water for 8 h. Then the seeds were put in an incubator for pre-germination for 24 h at 25 °C. The germinated seeds were sown in clean river sand and grown in a growth chamber. They were moistened with 1/2-strength Hoagland’s solution, and the condition of growth chamber was as followed: the light and dark cycle was 16 h/8 h, and temperature of the chamber was 28 °C of light/25 °C of darkness, a photosynthetic photon flux density of 500 µmol/m^2^/s and a relative humidity of 70%.

The 14-days-old seedlings were removed to sterile water to adapt to hydroponic conditions for 24 h. Then the roots of peanuts were soaked in PEG-6000 (20%) solution for 0 h (Control), 4 h, 8 h and 24 h. The antepenult leaves were sampled and frozen in liquid nitrogen immediately, then store at −80 °C. Three independent biological replicates (*n* = 3) were analyzed per treatment group. Each biological replicate consisted of 10 pooled plants grown in separate randomized blocks.

### Chlorophyll pigments contents

Chlorophyll (Chl) content was determined according to Lichtenthaler [14]. 0.1 g fresh leaves were homogenized by 10 mL ethanol (95%, v/v). The absorbance of the extract was taken at 665, 649, and 470 nm. The parameters were calculated using the following formulas: Chl a (mg g^–1^)=(13.95A665–6.88A649)×V/(1,000×W), Chl b (mg g^–1^)= (24.96A649–7.32 A665)×V/(1,000×W), carotenoids (Car) (mg g^–1^)=[(1,000 A470–2.05 Chl a–114.8 Chl b)/245]×V/(1,000 × W).

### Measurements of photosynthesis and chlorophyll fluorescence

The WUE was calculated as the formula of WUE = Pn/Tr, and the Pn, Tr were determined by CIRAS-2 (Hansatech, England). The leaf chamber conditions were as follows: a PPFD of 1200 µmol m^−2^ s^−1^, relative humidity of 70%, a leaf temperature of 25 °C, and a CO_2_ concentration of 380 µmol mol^−1^ in the leaf chamber. The leaves were dark-adapted for 30 min, then the fast chlorophyll fluorescence induction kinetic curve (OJIP transient) were performed using Pocket PEA (Hansatech, UK). The formulae and a glossary of terms in this study were showed in table S1. F_L_, F_K_ F_J_, and F_I_ present the fluorescence intensity at 150 µs (L-step), 300 µs (K-step), 2 ms (J-step) and 30 ms (I-step), the active fraction of oxygen evolving complex (OEC) centers and Q_A_-reducing centers (Q_A_-RC) were calculated according to Yang et al. [15] as the following formulas:

$$\mathrm{OEC}\;\mathrm{centers}={\left(1-{\mathrm V}_{\mathrm K}/{\mathrm V}_{\mathrm J}\right)}_{\mathrm{treatment}}/{\left(1-{\mathrm V}_{\mathrm K}/{\mathrm V}_{\mathrm J}\right)}_{\mathrm{control}}$$

$${\mathrm Q}_{\mathrm A-}\mathrm{RC}=\left[{\left(\mathrm{RC}/\mathrm{CS}\right)}_{\mathrm{treatment}}/{\left(\mathrm{RC}/\mathrm{CS}\right)}_{\mathrm{control}}\right]\;\left[{\left(\mathrm{ABS}/\mathrm{CS}\right)}_{\mathrm{treatment}}/{\left(\mathrm{ABS}/\mathrm{CS}\right)}_{\mathrm{control}}\right]$$

### Measurements of antioxidant system

The relative electrolyte leakage (EL) rate was tested by a conductivity meter (DDSJ − 308 F, Shanghai, China). The crude enzyme solution of antioxidant enzyme was extracted by phosphate buffer. Then the SOD (EC 1.15.1.1) crude enzyme mixture was reacted with nitro blue tetrazolium (750 µM), riboflavin (20 µM), L-methionine (150 mM) and sodium phosphate buffer (50 mM, pH = 7.8). The POD (EC 1.11.1.7) activity was measured according to Chen et al. [16], the 50 µL crude enzyme mixture was reacted with 0.95 mL of 0.2% guaiacol, 1 mL of 0.3% H_2_O_2_ and 1 ml of 50 mM phosphate buffer (pH = 5.5). The CAT (EC 1.11.1.6) activity was assayed according to Droillard et al. [17], the reaction mixture consisted of 50 mM sodium phosphate buffer (pH 7.0), 10 mM H_2_O_2_, 2 mM Na_2_-EDTA and crude enzyme solution.

### RNA‑seq library construction and transcriptomic data processing

Total RNA was extracted from 100 mg leaf tissues using TRIzol reagent (Invitrogen) following the manufacturer’s protocol. The high-quality RNA samples were used for cDNA library construction. The cDNA libraries were then constructed and sequenced using an Illumina high-throughput sequencing platform (Illumina, San Diego, California, USA). The clean data was alignment to the reference genome (https://www.peanutbase.org/data/public/Arachis_hypogaea/Tifrunner.gnm1.KYV3/arahy.Tifrunner.gnm1.KYV3.genome_main.fna.gz). The obtained sequences were uploaded to the NCBI BioProject database under the SRA accession number PRJNA657965. Gene expression was measured using FPKM, and differentially expressed genes were identified based on the threshold of |log2 Fold Change (FC)| ≥ 1.00 and FDR ≤ 0.05. Kyoto encyclopedia of genes and genomes (KEGG) pathway analysis was conducted using the KEGG database, while gene ontology (GO) functional enrichment and classification analyses were performed using the online tool agriGO.

### Statistical analysis

The variability (V) of peanut physiological trait was calculated by as the following formulas: V=(max-min)/max, and the △V=|V_NH5_-V_FH18_|. Statistical analysis was conducted using Microsoft Excel (Microsoft Corporation, USA) and SPSS 22 (SPSS Inc., USA), and the results were visualized using GraphPad Prism 8 (GraphPad Software, Inc.). Statistical significance among different time points was evaluated by one-way ANOVA, with the least significant difference (LSD) test employed for multiple comparisons.

## Results

### Transcriptomic analysis revealed regulation of photosynthesis related genes

To investigate the impact of drought stress on peanut, transcriptome analyses were conducted. The results revealed that a total of 14,126 and 8,982 genes were differentially expressed in FH18 and NH5, respectively, under drought stress conditions. Notably, a higher number of down-regulated genes were observed in the drought-sensitive variety (Fig. S1). Subsequently, these differentially expressed genes were subjected to GO pathway enrichment analysis, which showed that they were predominantly enriched in photosynthesis-related pathways in both varieties. In FH18, genes related to chlorophyll biosynthetic process (GO:0015995), photosystem II assembly (GO:0010207), and thylakoid membrane organization (GO:0010027) were enriched in the biological process (BP) category. In the cellular component (CC) category, terms such as chloroplast envelope (GO:0009941), chloroplast stroma (GO:0009570), photosystem II oxygen evolving complex (GO:0009654), chloroplast thylakoid lumen (GO:0009543), chloroplast thylakoid membrane (GO:0009535), and NAD(P)H dehydrogenase complex (plastoquinone) (GO:0010598) were enriched (Fig.S2 and Table S2). In NH5, GO terms related to photosynthesis were enriched in the BP category, including photosystem II assembly (GO:0010207), chlorophyll catabolic process (GO:0015996), and photosynthetic electron transport in photosystem I (GO:0009773). In the CC category, terms such as chloroplast envelope (GO:0009941), chloroplast thylakoid membrane (GO:0009535), chloroplast thylakoid lumen (GO:0009543), photosystem II oxygen evolving complex (GO:0009654), chloroplast thylakoid (GO:0009534), chloroplast stroma (GO:0009570), and photosystem I reaction center (GO:0009538) were enriched (Fig. S2 and Table S3).

**Fig. 1:**
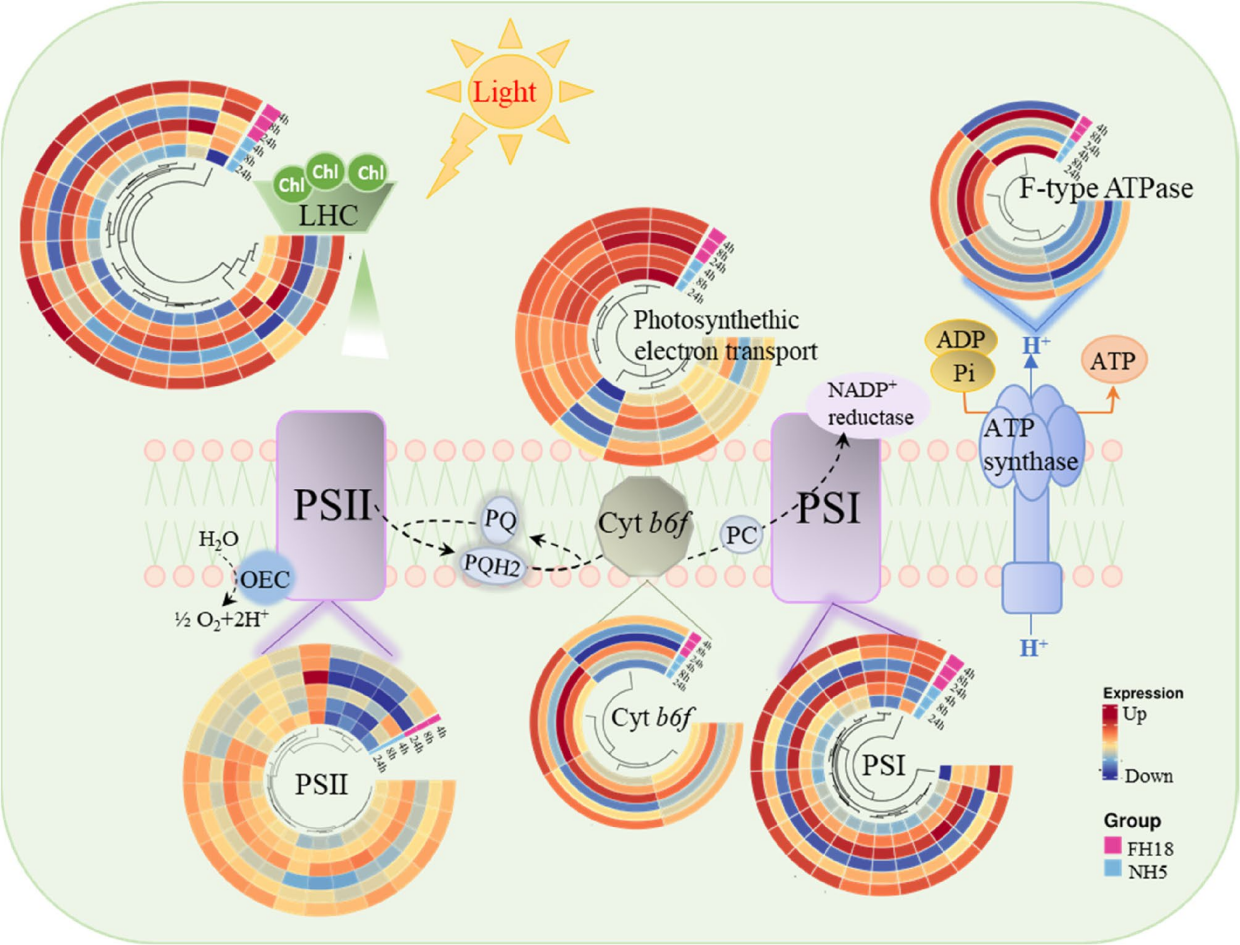
Schematic representation of photosynthetic electron transport chain and associated light reactions​​. This diagram illustrates key components of the light-dependent reactions in chloroplasts and expression patterns of related genes. The OEC at PSII catalyzes water splitting while PQ shuttles electrons through the Cyt b6f complex to PC. LHC transfer excitation energy to reaction centers. F-type ATP synthase facilitates proton gradient-driven ATP synthesis. Color-coded expression patterns in the lower panel indicate: red​​: upregulated, blue: downregulated

Subsequently, the transcriptional regulation of photosynthesis genes in peanut was explored based on the KEGG pathways. Figure 1 and Table S4 illustrates that a significant number of genes involved in photosynthesis and photosynthesis-antenna proteins were specifically differentially expressed in response to drought stress, most of these genes, such as genes encoding psbS (photosystem II 22 kDa protein, arahy.L9FPHH, arahy.QP7RRL), PsbQ (PsbQ-like protein 1, arahy.T46WBJ, arahy.TVDX40, arahy.I21UC1, arahy.IUT8LB, arahy.I4CVDG), and PsbP (oxygen-evolving enhancer protein, arahy.G1SUYP, arahy.Y7HUGW, arahy.1944SK), were downregulated. The decrease was more pronounced in FH18 (Fig. 1), indicating a more severe interference in the drought-sensitive variety.

### Effect of drought stress on photosynthetic pigment content and net photosynthetic rate

**Fig. 2:**
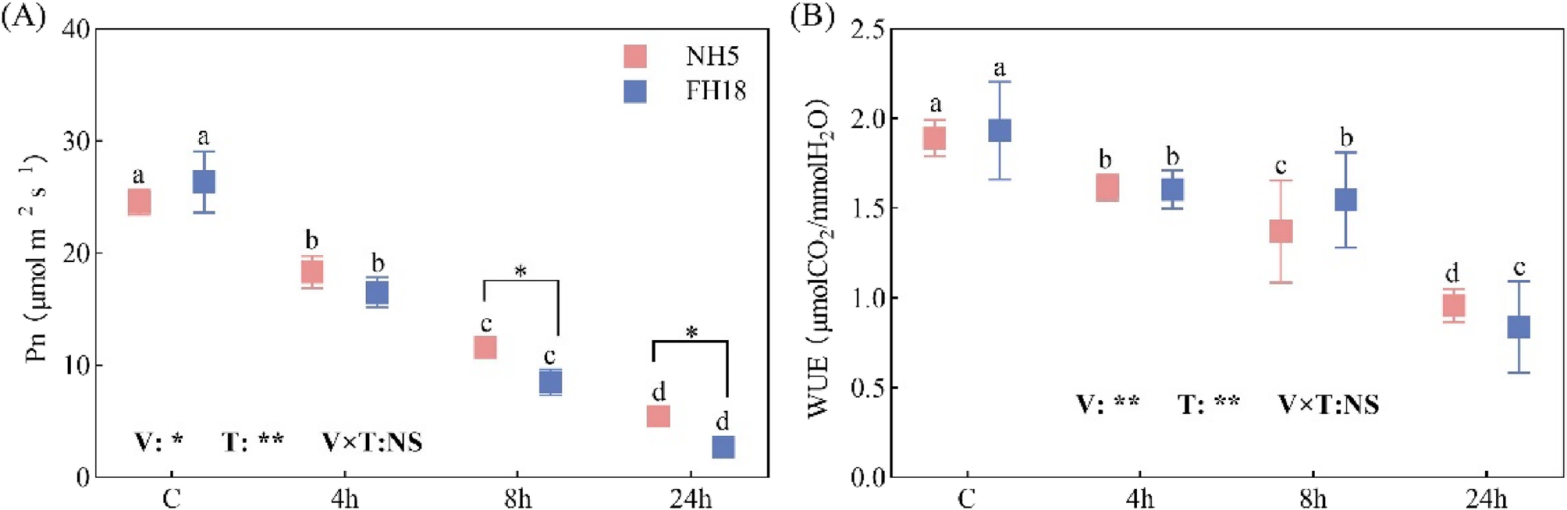
The effect of drought stress on net photosynthetic rate (Pn, A) and water use efficiency (WUE, B) of drought tolerant variety (NH5) and drought sensitive variety (FH18). Different lowercase letters indicate significant differences among different treatments (*P* < 0.05). * and ** significant difference at the 0.05 and 0.01 probability level; NS no significant difference

With the extension of drought stress, the relative water content of peanut leaves gradually decreases, and significant differences emerged between the two varieties at 8 h. By observing the plant phenotype, it was found that the main stem of FH18 was dry and curved (Fig S3). Then, the photosynthetic performances of peanut were further evaluated under drought stress. Table 1 shows the changes in photosynthetic pigments. In NH5, the contents of Chl a, Chl b, Chl a + b and Car increased by 51.6%, 170.0%, 88.6%, and 50.0%, respectively, after 24 h of drought stress compared to the control. However, in FH18, the pigments increased significantly at 8 h but then decreased by 14.4%, 52.1%, 30.9% and 13.1% for Chl a, Chl b, Chl a + b and Car, respectively, at 24 h. It is evident that the interaction between variety and treatment significantly affected the levels of Chl a, Chl b, Chl a + b, and the chlorophyll a/b ratio. Concurrently, as showed in Fig. 2, both NH5 and FH18 experienced a steep decline in Pn and WUE, reaching their lowest points at 24 h of drought stress.

**Table 1:** Effect of drought stress on the photosynthetic pigment content

		Chl a (mg g^−1^)	Chl b (mg g^−1^)	Chl a + b (mg g^−1^)	Chl a/b	Car (mg g^−1^)
NH5	C	0.93 ± 0.02c	0.40 ± 0.01c	1.32 ± 0.04c	2.34 ± 0.04a	0.28 ± 0.01c
	4 h	1.16 ± 0.12b	0.57 ± 0.09bc	1.73 ± 0.21b	2.06 ± 0.18a	0.35 ± 0.04b
	8 h	1.24 ± 0.12ab	0.59 ± 0.05b	1.83 ± 0.16b	2.10 ± 0.05a	0.36 ± 0.03b
	24 h	1.41 ± 0.07a	1.08 ± 0.14a	2.49 ± 0.18a	1.32 ± 0.17b	0.42 ± 0.01a
FH18	C	0.90 ± 0.19b	0.37 ± 0.08b	1.27 ± 0.17c	2.450.05a	0.29 ± 0.06b
	4 h	1.17 ± 0.06ab	0.56 ± 0.04b	1.73 ± 0.09b	2.11 ± 0.07a	0.36 ± 0.02ab
	8 h	1.25 ± 0.06a	0.98 ± 0.03a	2.23 ± 0.24a	1.38 ± 0.13b	0.38 ± 0.01a
	24 h	1.07 ± 0.19ab	0.47 ± 0.05b	1.54 ± 0.12bc	2.35 ± 0.11a	0.33 ± 0.04ab
Variation source						
Variety (V)		NS	NS	NS	NS	NS
Treatment (T)		**	**	**	**	**
V×T		NS	**	**	**	NS

The different small letters within the same column mean significantly different at *P* < 0.05. *and ** significant difference at the 0.05 and 0.01 probability level; NS no significant difference

### Effect of drought stress on antioxidant system

**Fig. 3:**
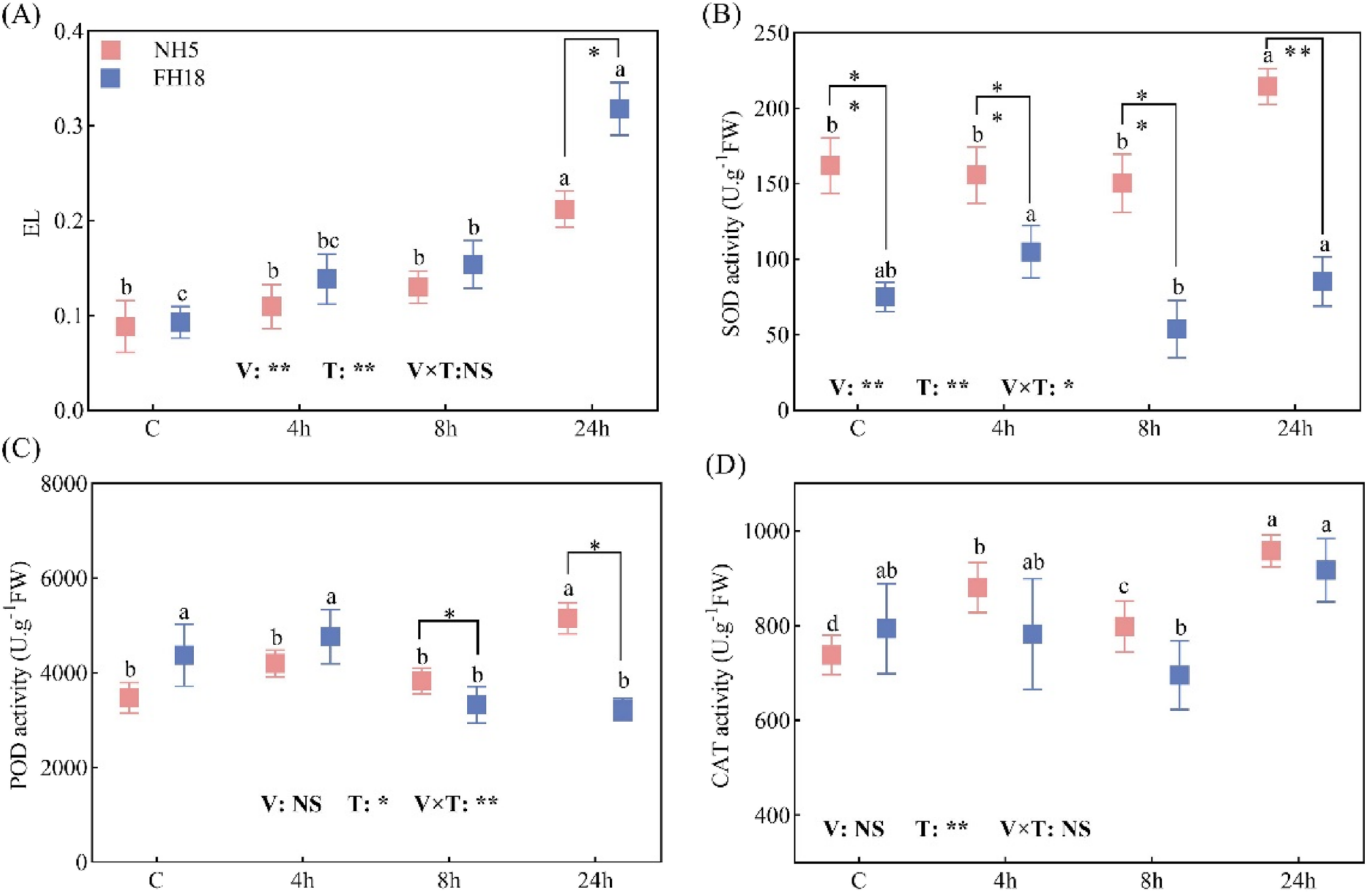
The effect of drought stress on membrane damage and antioxidant capacity of drought tolerant variety (NH5) and drought sensitive variety (FH18). **A** relative electrolyte leakage rate, (**B**) superoxide dismutase activity, (**C**) peroxidase activity, (**D**) catalase activity. Different lowercase letters indicate significant differences among different treatments (*P* <0.05). *and ** significant difference at the 0.05 and 0.01 probability level; NS no significant difference

Drought stress caused significant lipid peroxidation in both peanut genotypes, though the extent of damage differed markedly between the tolerant (NH5) and sensitive (FH18) varieties (Fig. 3). Statistical comparisons revealed significantly higher EL rates in FH18 compared to NH5 at 24 h (*p* < 0.05). Specifically, the EL rate of NH5 increased by 23.4%, 46.6% and 138.6% compared with the control, whereas FH18 exhibited more severe membrane damage with EL rate increments of 49.0%, 65.4% and 242.3% under progressive drought stress. The antioxidant response patterns also showed statistically significant genotypic differences, NH5 maintained significantly higher enzymatic activity levels than FH18 throughout the stress period. In NH5, SOD and POD activities showed robust increases of 32.3%, 48.4% and 29.8% respectively at 24 h, which significantly greater than the corresponding values in FH18. Conversely, FH18 displayed a compromised antioxidant response with only 13.6% and 15.5% increases in SOD and CAT activities, accompanied by a 20.5% decrease in POD activity.

### Effect of drought stress on chlorophyll fluorescence properties

**Fig. 4:**
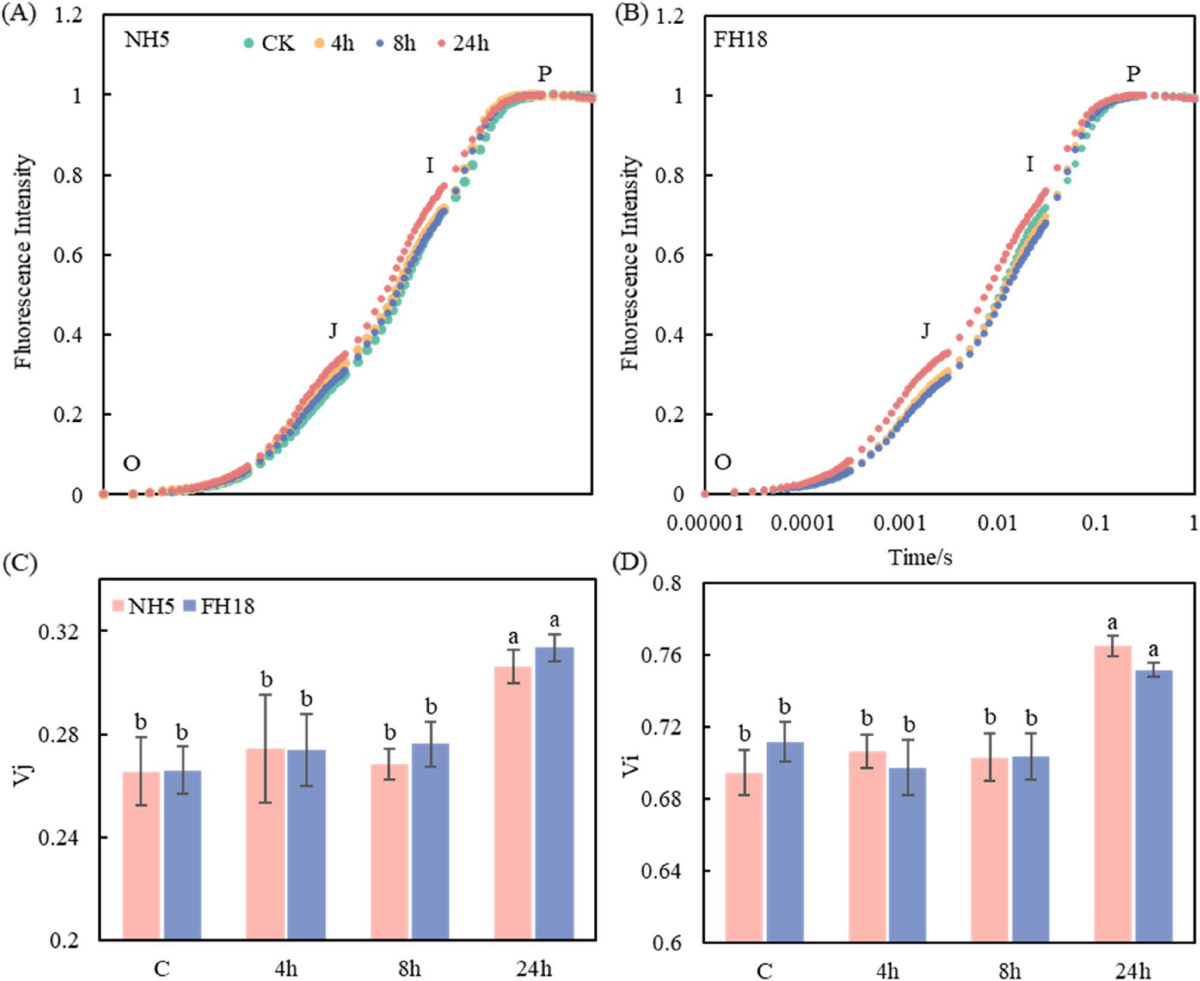
Chl a fluorescence rises OJIP curves of drought tolerant variety (NH5) and drought sensitive variety (FH18) under drought stress. Different lowercase letters indicate significant differences among different treatments (*P* < 0.05)

To investigate the mechanism of photosynthetic rate under drought stress, the fluorescence rise OJIP curves of peanut plants were measured. As shown in Fig. 4, the J-phase increased with the extension of drought treatment in peanuts, especially in FH18 at 24 h treatment. A similar trend was observed in the I-phase with a significantly increased at 24 h. The V_j_ was not changed obviously at 4 h and 8 h treatment, while significantly increased at 24 h by 15.3% and 17.9% in NH5 and FH18, respectively. Likewise, the V_i_ significantly increased at 24 h by 10.1% and 5.7% in NH5 and FH18.

**Fig. 5:**
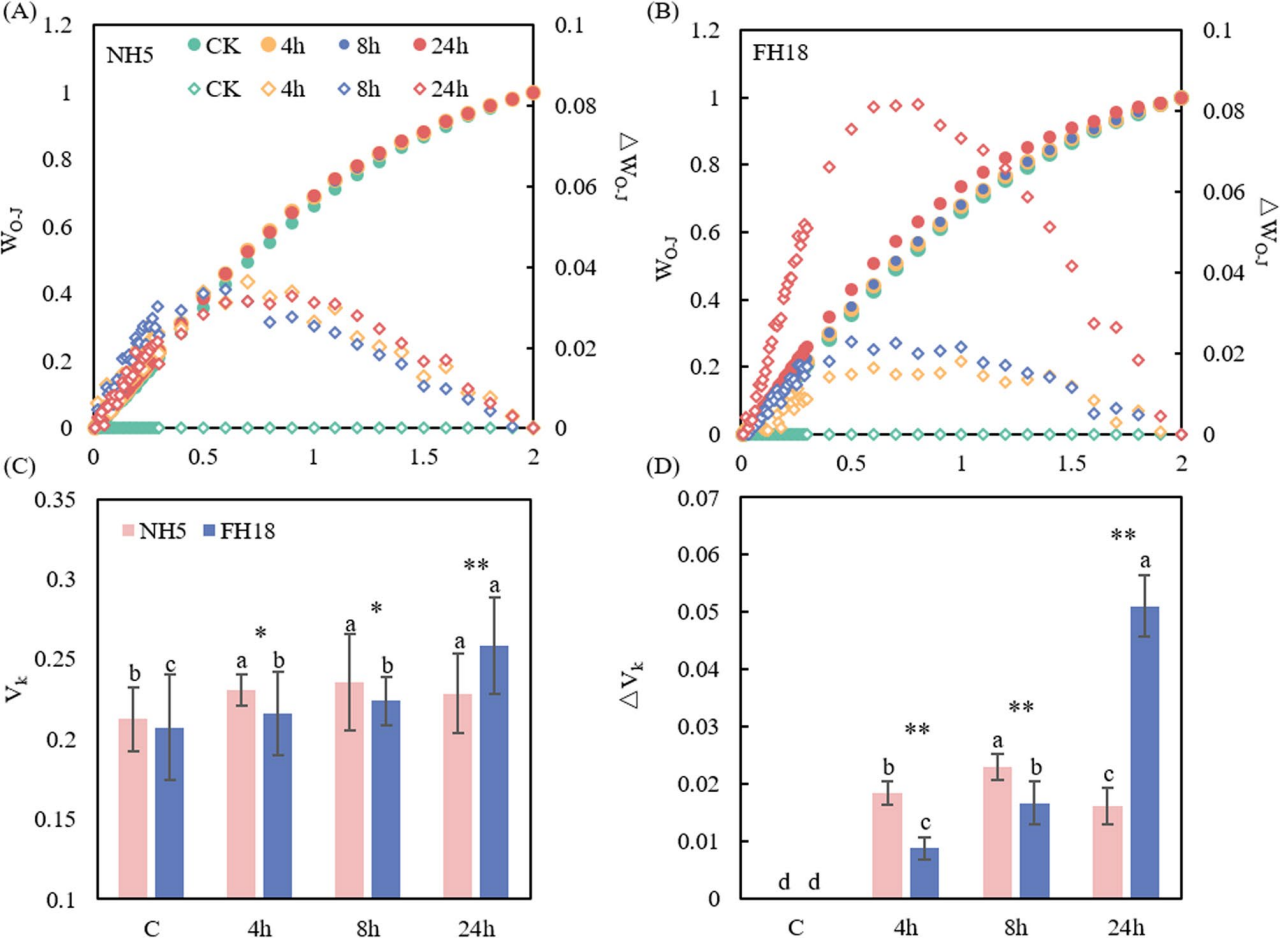
Effect of drought stress on the K-step of the OJIP curves of drought tolerant variety (NH5) and drought sensitive variety (FH18). The W_OJ_=(F_t_–F_O_)/(F_J_–F_O_), and △W_OJ_=W_OJ(treatment)_- W_OJ(control)_. Different lowercase letters indicate significant differences among different treatments (*P* < 0.05)

Furthermore, we analyzed the O-J phase for the K step by double standardization (Fig. 5A and B). As we can see, the K step increased after drought stress, and there was no significant difference between curves at 4–24 h treatment in NH5. However, the curve of FH18 sharply increased at 24 h. Then, relative variable fluorescence at the K step was calculated and normalized to quantify changes in the K step in OJIP curves (Fig. 5C and D). The results showed that the V_k_ and △V_k_ of FH18 exhibited significantly higher levels by the increase of 24.6% at 24 h.

**Fig. 6:**
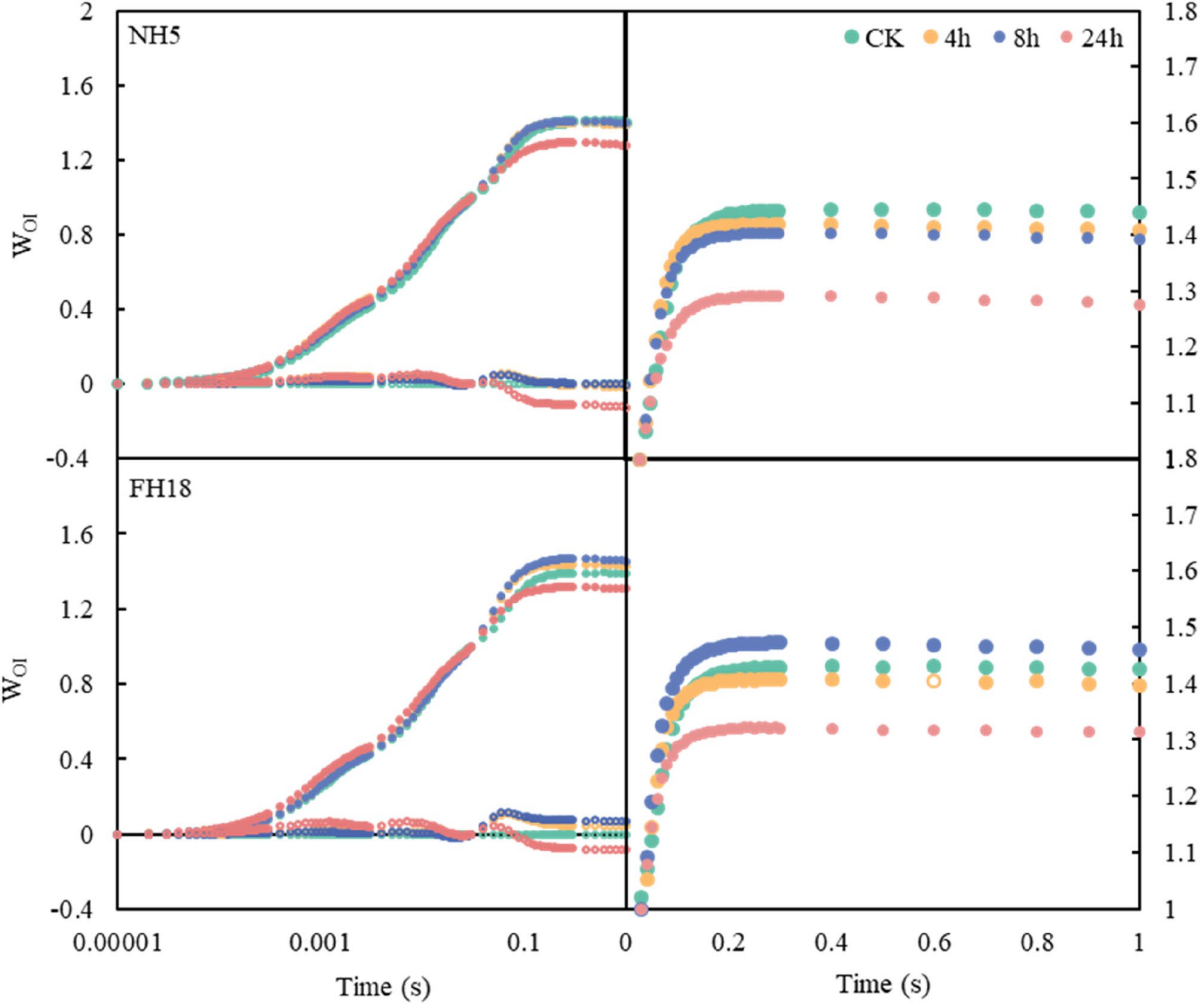
Effect of the fluorescence O–I phase in drought tolerant variety (NH5) and drought sensitive variety (FH18) under drought stress. W_OI_=(F_t_-F_o_)/(F_I_-F_o_), ΔW_OI_=W_OI(treatment)_-W_OJ(control)_

Subsequently, the analysis of the O-I phase was performed. The overall trends of the O–I phase of the two peanut varieties were generally consistent (Fig. 6). The amplitude of W_OI_≥1 fraction decreased at 24 h both in NH5 and FH18, which indicated that the terminal electron acceptor pool on the PSI receptor side has been disrupted by drought stress.

### Analysis of the JIP-test parameters

**Fig. 7:**
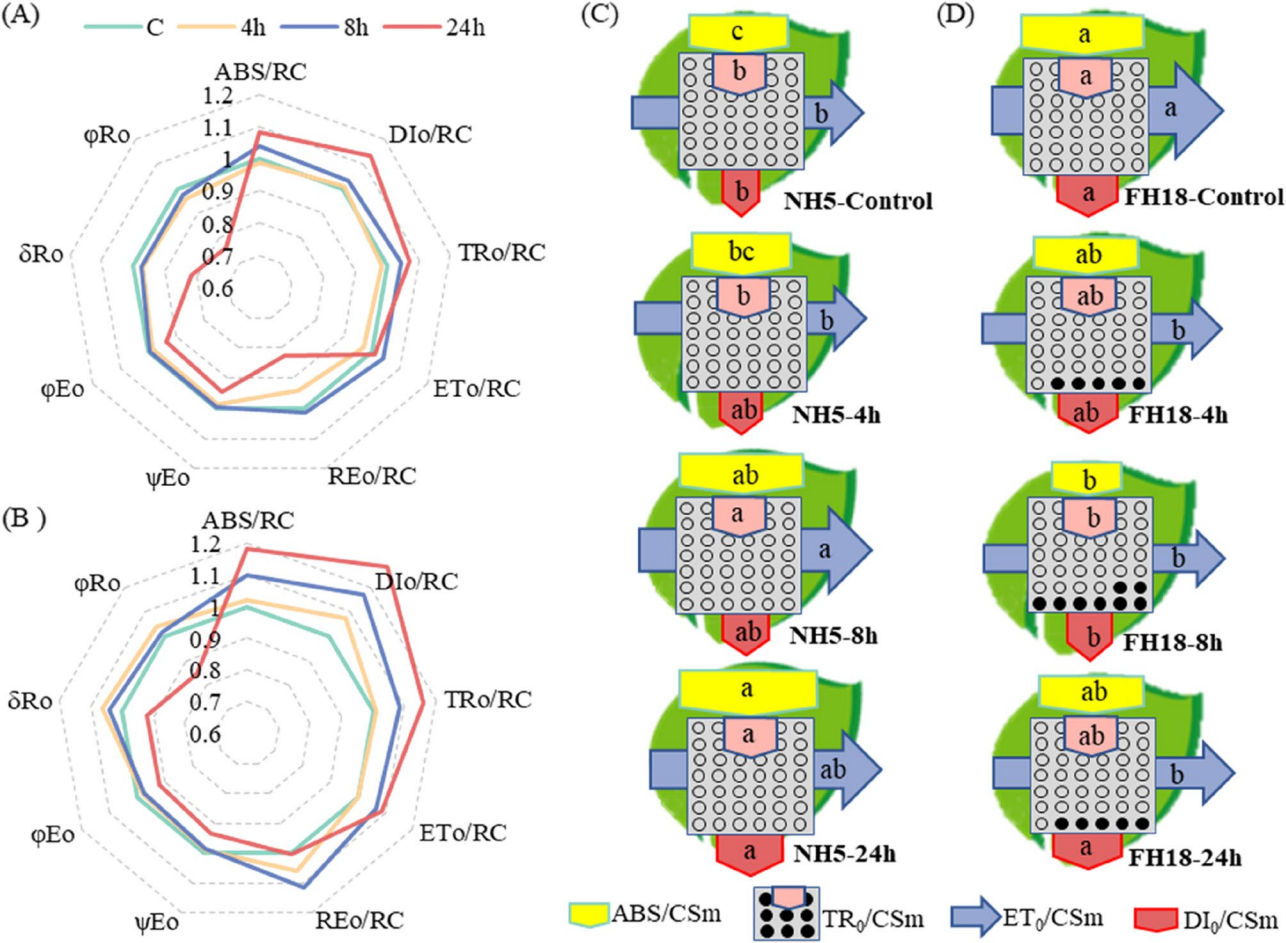
Relative changes of OJIP test parameter and pipeline models of phenomenological energy fluxes per excited cross section of drought tolerant variety (NH5) and drought sensitive variety (FH18) under drought stress. A and C: NH5, B and D: FH18, and different lowercase letters in pipeline models indicate significant differences among different treatments (*P* < 0.05)

The specific energy flux per active PSII reaction center of peanuts was determined in this study. The absorbed photon flux per active PSII (ABS/RC), dissipated energy (as heat and fluorescence) flux per active PSII (DI_0_/RC) and trapped energy flux per active PSII (TR_0_/RC) in NH5 showed a slight difference at 4 h and 8 h compared with the control, while significantly increased by 8.1%, 13.6% and 7.2% at 24 h. In FH18, the ABS/RC, DI_0_/RC and TR_0_/RC continually increased under drought stress, and significant increases could be observed at 24 h by 18.1%, 28.2% and 16.3%. The electron flux from Q_A_^‒^ to the plastoquinones pool per active PSII (ET_0_/RC) had no significant change in NH5 under drought stress, while increased significantly in FH18 at 8 h and 24 h by 6.9% and 8.7%. Besides, electron flux from Q_A_^‒^ to the final electron acceptors of PSI per active PSII (RE_0_/RC) significantly reduced by 17.5% in NH5 at 24 h, however, in FH18, the RE_0_/RC increased by 5.8% and 11.2% at 4 h and 8 h (Fig. 7A and B). Additionally, as shown in the pipeline models (Fig. 7C), the absorption flux per cross section (ABS/CSm), trapped flux per cross section (TR_0_/CSm), electron transport flux per cross section (ET_0_/CSm) and thermal dissipation energy flux per cross section (DI_0_/CSm) gradually increased in NH5. In contrast, the TR_0_/CSm and ET_0_/CSm in FH18 were marked decrease by 13.4% and 14.6% under drought stress.

The results of quantum yield and efficiencies showed that there were no significant changes in quantum yield of electron transport from Q_A_^⁻^ to Q_B_ (ψEo), quantum yield for electron transport (φEo), reduced intersystem electron acceptors to the final electron acceptors of PSI (δRo) and quantum yield for reduction of the end electron acceptors at the PSI acceptor side (φRo) at 4 h and 8 h of both NH5 and FH18 (Fig. 7A and B). With the duration of drought stress, ψEo, φEo, δRo and φRo significantly decreased by 5.4%, 6.4%, 19.0 and 23.1% in NH5 and 6.5%, 7.8%, 7.8% and 15.1% in FH18 at 24h.

**Fig. 8:**
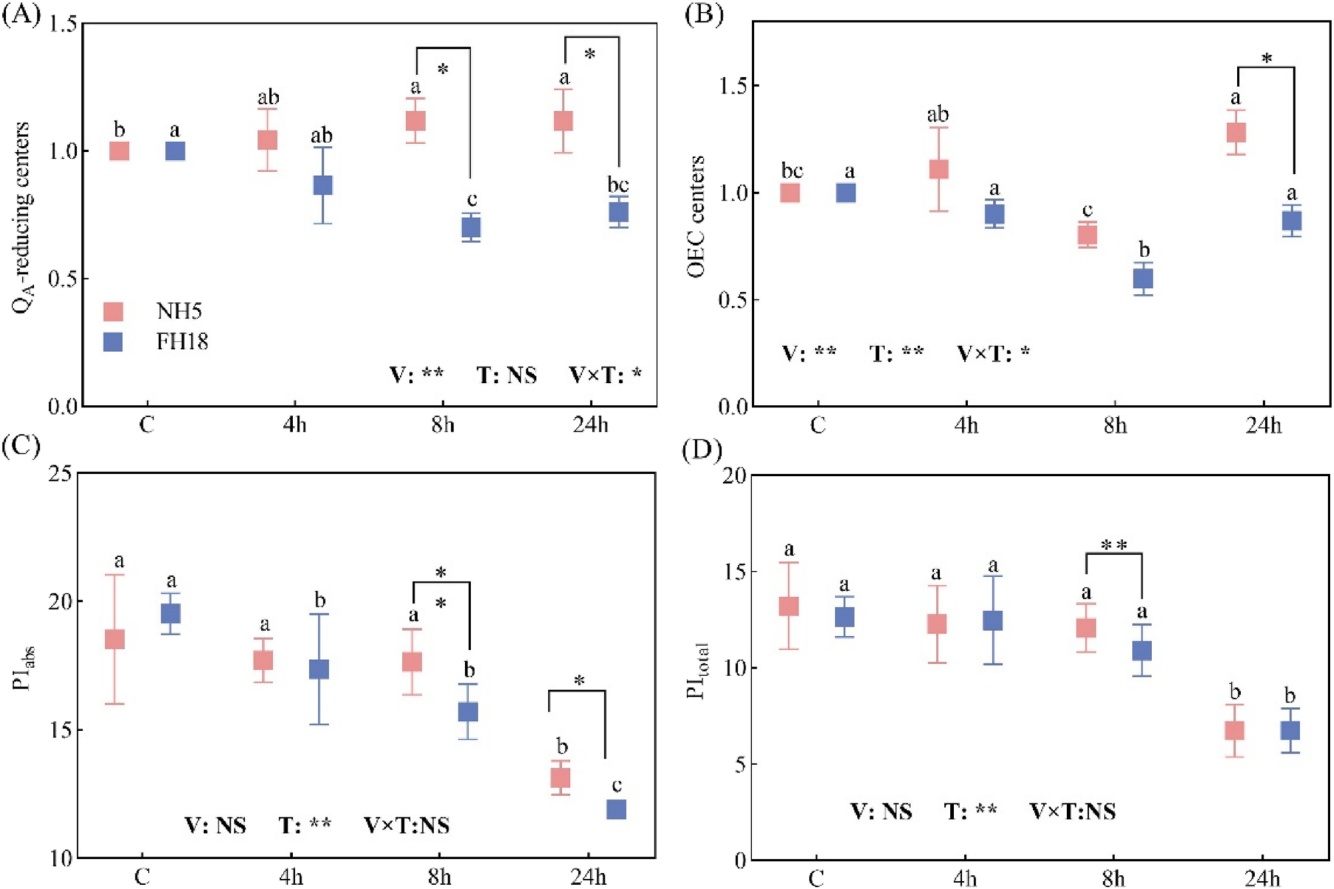
Effects of drought stress on the JIP-test parameters of drought tolerant variety (NH5) and drought sensitive variety (FH18). Different lowercase letters indicate significant differences among different treatments (*P* < 0.05). * and ** significant difference at the 0.05 and 0.01 probability level; NS no significant difference

The Q_A_-RC parameter exhibited contrasting patterns, with NH5 showing a 11.7% increase at 24 h, while FH18 declined to 76.1% of control levels. The OEC centers of both NH5 and FH18 suffered acute damage at 8 h by 26.3% and 42.2% compared with control. But differential recovery capacity emerged by 24 h, and NH5 reached a higher value that significantly higher than FH18 (*p* < 0.05). Performance indexes further corroborated varietal divergence. PI_abs_ degradation initiated earlier in FH18, showing significant reduction preceding NH5’s response. By 24 h, PI_abs_ losses reached 29.1% in NH5 versus 39.1% in FH18. Besides, the PI_total_ did not change significantly at 4 h and 8 h in peanuts, while distinctly decreased at 24 h by 49.1% and 46.7% in NH5 and FH18 (Fig. 8).

### Peanut variability and correlation analysis

**Fig. 9:**
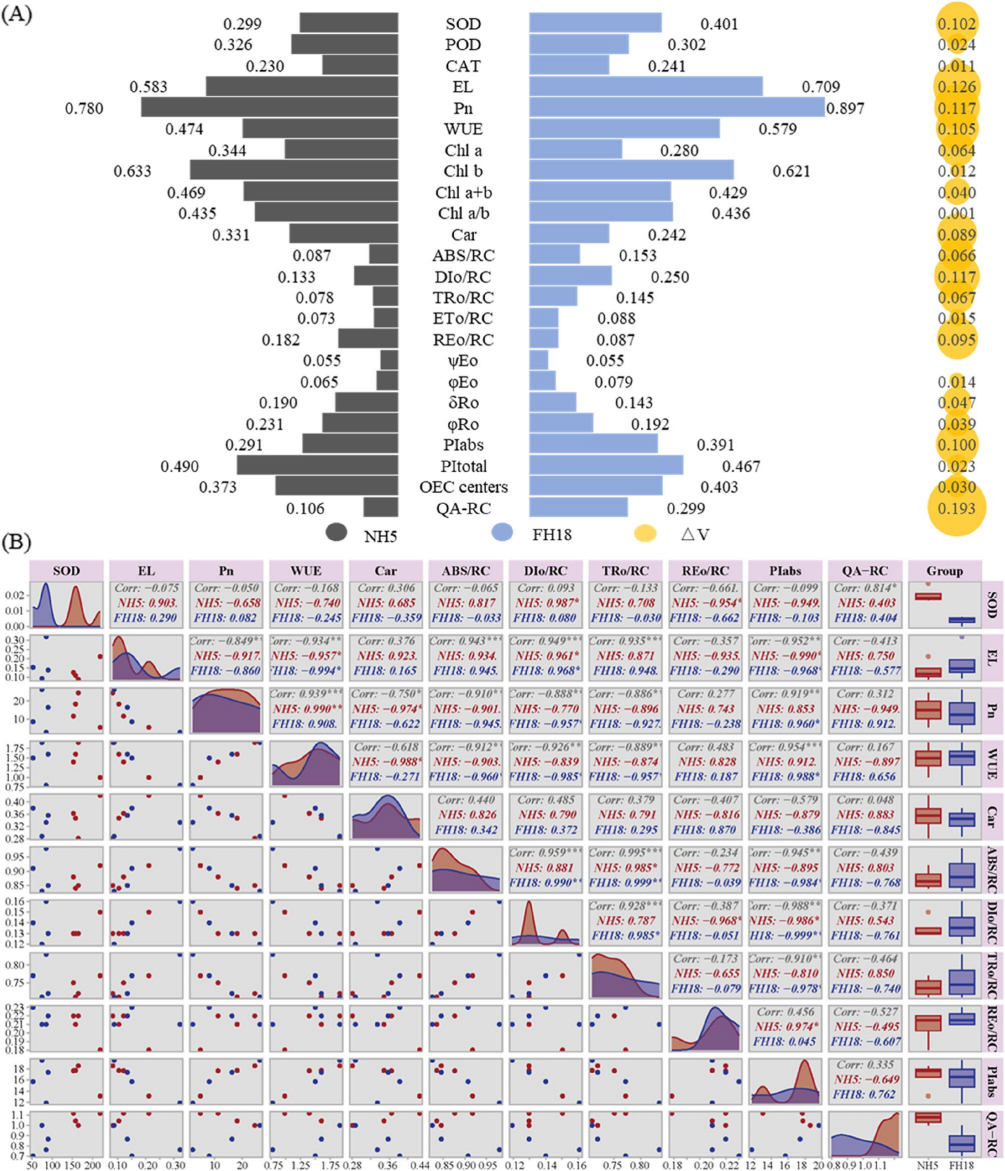
Variability and correlation analysis of peanut with different drought tolerance. **A** The variability of peanut, △V=|variability_NH5_- variability_FH18_|. **B** The correlation analysis of peanut, *p*-value < 0.001 ***, *p*-value < 0.01 **, *p*-value < 0.05 *, *p*-value < 0.10

As illustrated in Fig. 9A, a comparative variability analysis was conducted between drought-tolerant and drought-sensitive peanut varieties. Both NH5 and FH18 exhibited substantial variation values in EL (0.583 and 0.709), Pn (0.780 and 0.897), and Chl b (0.633 and 0.621), suggesting considerable disruption by drought stress. Furthermore, eleven traits demonstrated △V values exceeding 0.07 between the peanut varieties, highlighting significant differences in their response to drought stress. Notably, Q_A_-RC exhibited the highest △V value of 0.19, while PI_abs_, DIo/RC, REo/RC, ABS/RC, and TRo/RC also showed marked differences with the △V ranged from 0.066 to 0.1. These findings indicate wide variations in the PSII reaction center activity of the two varieties under drought stress.

Then, the eleven traits exhibiting higher △V values underwent correlation analysis (Fig. 9B). The EL, ABS/RC, DI_0_/RC and TR_0_/RC were significantly positively with each other, while they were significantly and negatively correlated with PI_abs_ and Pn. Notably, Pn showed a strong positive correlation with both WUE and PI_abs_. Additionally, WUE was negatively correlated with EL, and Q_A_-RC displayed a unique positive correlation solely with SOD. Marked variations were evident in the correlations observed among the indices of the different peanut varieties.

## Discussion

Drought stress constitutes a persistent threat to global crop productivity, with climate models projecting increasing aridity severity in peanut cultivation regions [1]. Previous studies have shown that drought stress at the seedling stage has deleterious impacts on photosynthesis and seedling establishment [18].

The photosynthetic apparatus emerges as a primary physiological constraint under drought conditions, exhibiting particular susceptibility to water deficit across plant species. Turning now to the experimental evidence, the GO analysis in the present study showed that the DEGs were significantly enriched in multiple pathways related to photosynthesis. In our previous study, the KEGG analysis also showed that DEGs of peanut under drought stress were enriched in pathways related photosynthesis. These observations are reinforced through weighted gene co-expression network analysis, where three core modules show striking correlations with chlorophyll fluorescence parameters [19], extending earlier observations of drought-responsive transcriptional networks in peanut. Among the down-regulated genes, we identified multiple PsbP and PsbQ homologs. The N-terminal domain of PsbP exhibits regulatory control over the redox potential of cytochrome b_559_ within the PSII complex [20]. This redox modulation capability enables PsbP to participate in fine-tuning electron transport dynamics. These proteins were found to play pivotal roles in maintaining the structural integrity of PSII complexes [21]. Despite not being directly involved in the water-splitting reaction, PsbP and PsbQ proteins crucially contribute to stabilizing the OEC architecture. This stabilization ensures efficient energy transduction while providing photoprotective mechanisms against oxidative damage.

The PSII complex incorporates a peripheral light-harvesting antenna system, comprising chlorophyll a/b and carotenoids, collectively optimize light absorption and energy transfer to the reaction center [5]. Drought stress typically suppresses chlorophyll biosynthesis by impairing rate-limiting enzymes, ultimately depleting chlorophyll reserves [22]. Contrary to this paradigm, NH5 exhibited an 88.6% chlorophyll accumulation under drought, contrasting sharply decline with FH18. This anomalous response aligns with observations in drought-tolerant blue honeysuckle, where chlorophyll content transiently increases under mild drought [23]. The divergent behavior of NH5 suggests two potential drought adaptation mechanisms: (1) sustained activation of chlorophyll anabolic pathways, and/or (2) effective suppression of chlorophyllase-mediated catabolism through antioxidative systems (SOD, POD and CAT upregulation). Such homeostasis may synergistically enhance PSII photoprotection while maintaining light-harvesting efficiency under water deficit.

Photoinhibition-induced blockage of electron transport (particularly at the Q_A_ to Q_B_ stage) leads to excessive reducing power accumulation, forcing molecular oxygen to act as an alternative electron acceptor [24], and this process drives massive generation of ROS [25]. The constant generation of ROS causes continuous oxidative stress, resulting in polyunsaturated fatty acid peroxidation [26]. This phenomenon may assailant the stability of cellular membranes, ultimately leading to leakage and losses of intracellular materials [27]. Drought stress marked increased the EL in peanut (Fig. 3 A), and significantly higher levels of variability could be observed in FH18 (Fig. 9A). It demonstrated that drought stress has broken the balance of ROS due to the excessive excitation pressure, which alerts the cell membrane permeability, especially in FH18.

The classical scheme for photoinhibition posits that excessive ROS directly attacks the PSII reaction center, while simultaneously inhibiting PSII repair mechanisms [28]. Therefore, the OJIP-fluorescence parameters were performed to investigate the physiological status of photosynthetic apparatus under drought stress. The pronounced increase in J-step fluorescence intensity in FH18 (Fig. 4) reflects ​​impaired electron transfer from Q_A_ to Q_B_ [29]​​. Furthermore, we normalized the O-J phase which reflected the association with drought stress of K bands in peanuts. Given already published literature, the significant rise of V_k_ and △V_k_ in FH18 suggested the inactivation of the OEC, which impedes the transfer of electrons from the donor to the acceptor side of PSII [30, 31]. Consistent with this finding, the same phenomenon was observed in mung bean, particularly in drought-sensitive variety [32]. Studies have revealed that a smaller amplitude of W_OI_≥1 fraction indicating a smaller terminal electron receptor pool on the PSI receptor side [33]. The significant decline of W_OI_≥1 fraction in this study indicated the decreasing size of the terminal electron acceptor pool on the PSI receptor side in two varieties under drought stress.

Additionally, Fig. 7 reveals that there has been a steady increase in the value of ABS/RC, TR_0_/RC and ET_0_/RC, and higher levels could be observed in drought sensitive variety. ABS/RC was used as indicator to reflect the functional antenna size [34]. Plants are capable of adapting to changes in their environment by adjusting their effective antenna size. Under drought stress, the increase of ABS/RC suggested the reduce of effective antenna size and absorbed energy [35]. While the TR_0_/CSm and ET_0_/CSm were downregulating in FH18 (Fig. 7C). A possible explanation for this phenomenon is that FH18 was unable to effectively regulate the reaction centers of PSII during drought stress, resulting in damage to these centers. It has been widely observed that in drought-sensitive varieties, PSII reaction centers are damaged under excessive excitation pressure [32, 35].

The PI_abs_ serves as a comprehensive indicator integrating several fundamental photosynthetic processes including light energy absorption, excitation energy trapping, and conversion efficiency of excitation energy to electron transport. This multiparametric nature makes PI_abs_ particularly responsive to physiological perturbations, as any functional alteration in these constituent processes directly impacts its quantitative value [36]. Notably, PI_abs_ demonstrates enhanced sensitivity to drought stress compared to conventional photosynthetic parameters [37]. Statistical validation through principal component analysis in sour cherry study revealed statistically significant correlations between PI_abs_/PI_total_ values and drought stress intensity [38]. This diagnostic capacity has been further corroborated in related species, where PI_abs_ and PI_total_ measurements have proven effective for evaluating drought tolerance mechanisms in commercial cultivars of sweet cherry and apple [39, 40]. In this study, the PI_abs_ of both NH5 and FH18 declined sharply under 24 h-drought stress, ​​with the drought-sensitive variety (FH18) exhibiting a more pronounced reduction (Fig. 8C). This aligns with studies in sesame where PI_abs_ served as a screening marker for drought tolerance [41]. The decline in PI_abs_ and PI_total_ (which reflects limitations on both PSII activity and PSI structure/function indicates a systemic disruption in photosynthetic energy absorption, trapping, and electron transport [42, 43]. Notably, the differential stability of PSII reaction centers between varieties (evidenced by variations in DI_0_/RC, RE_0_/RC, ABS/RC, and TR_0_/RC) may be mechanistically linked to their antioxidant capacity. For instance, the drought-tolerant variety NH5 showed both higher antioxidant enzyme activity and less severe PI_abs_ inhibition, thereby preserving photochemical efficiency. ​​Conversely, the sensitive variety FH18 exhibited lower antioxidant enzyme activity alongside greater PI_abs_ suppression, implying a feedback loop where accumulated ROS exacerbates photoinhibition. ​​These observations highlight that drought tolerance involves not only photosynthetic apparatus resilience but also dynamic coordination between antioxidant defense and photochemical recovery pathways.

## Conclusion

Our findings demonstrate that drought stress severely compromises photosynthetic efficiency through coordinated molecular and physiological perturbations. While both cultivars exhibited downregulation of photosynthesis-related genes and impaired electron transport between Q_A_ to Q_B_, the differential resilience of PSII reaction centers emerged as a critical determinant of drought tolerance. Crucially, the cultivar-specific resilience of PSII reaction centers, particularly in maintaining Q_A_ to Q_B_ electron transfer continuity and OEC stability, directly correlated with their differential drought tolerance phenotypes. These results position PSII reaction center dynamics as a central regulatory hub in drought adaptation, providing functional markers for screening drought-tolerant germplasm and informing targeted protection strategies for photosynthesis under water deficit.

## Supplementary Information

Additional file 1: Fig S1. Differentially expressed genes in peanut under drought stress. Fig S2. GO annotation of differentially expressed genes in peanut under drought stress. Fig S3. Changes in the phenotype of peanut plants and the relative water content of leaves under drought stress.

Additional file 2: Table S1. The normalization of chlorophyll a fluorescence transient and relative parameters specific fluxes per active PSII reaction center. Table S2. GO annotation of differentially expressed genes in FH18. Table S3. GO annotation of differentially expressed genes in NH5. Table S4 Differentially expressed genes involved in photosynthesis in peanut.

## Data Availability

The sequencing data are available in the NCBI Sequence Read Archive (SRA) under accession number PRJNA657965.
