# Supplementary material for: Differential photosynthetic responses to drought stress in peanut varieties: insights from transcriptome profiling and JIP-Test analysis
Source: BMC Plant Biol. 2025 Jul 25;25:957. doi: 10.1186/s12870-025-06984-y (PMC12291314; doi:10.1186/s12870-025-06984-y)
Supplement: Supplementary file 1 — Additional file 1: Fig S1. Differentially expressed genes in peanut under drought stress. Fig S2. GO annotation of differentially expressed genes in peanut under drought stress. Fig S3. Changes in the phenotype of peanut plants and the relative water content of leaves under drought stress. [file 12870_2025_6984_MOESM1_ESM.docx]

**Differential Photosynthetic Responses to Drought Stress in Peanut Varieties: Insights from Transcriptome Profiling and JIP-Test Analysis**

Jingyao Ren^1,2^, Pei Guo^2^, Xin Ai^2^, Xinlei Ma^2^, Jing Wang^2^, Xinhua Zhao^2^, Hongtao Zou^1^*, Haiqiu Yu^2,3^*

1. College of Land and Environment, Shenyang Agricultural University, Shenyang, China

2. College of Agronomy, Shenyang Agricultural University.Shenyang, China

3. Liaoning Agriculture Vocational and Technical College, Yingkou, China

*Corresponding author:

E-mail: hongtaozou208@163.com (H. Zou), [yuhaiqiu@syau.edu.cn](mailto:yuhaiqiu@syau.edu.cn) (H. Yu)


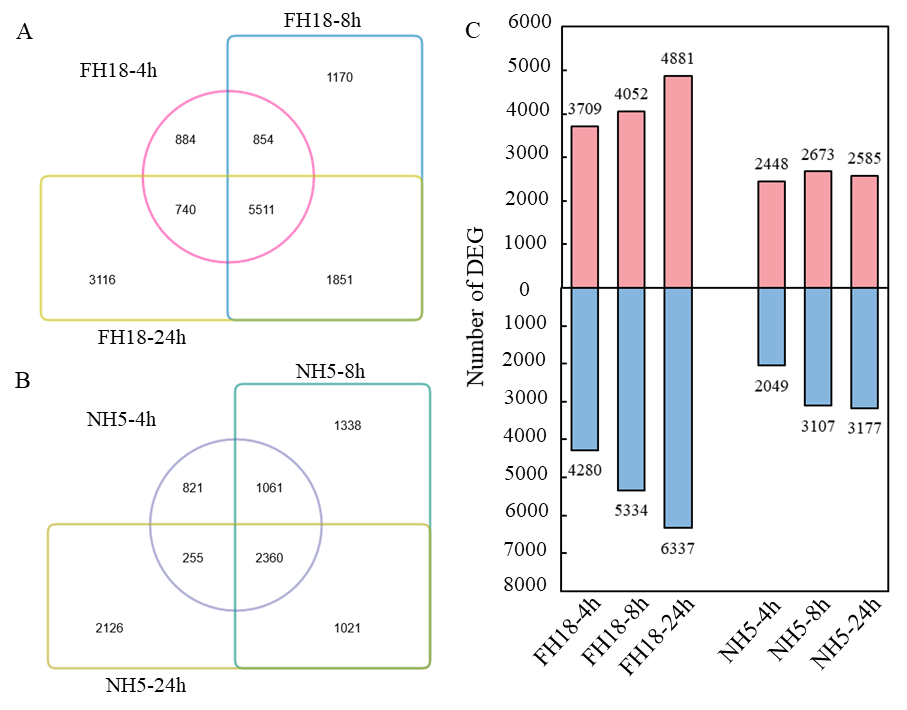


Fig.S1 Differentially expressed genes in peanut under drought stress


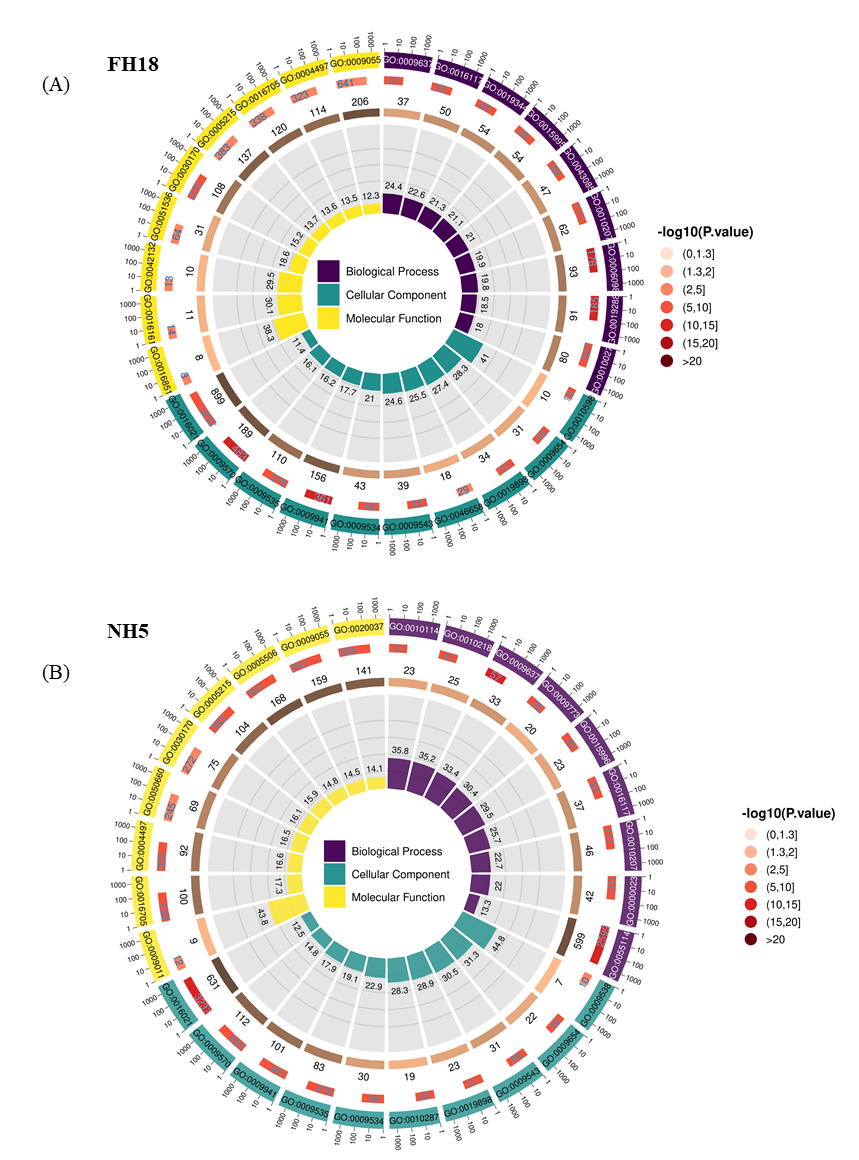


Fig.S2 GO annotation of differentially expressed genes in peanut under drought stress.


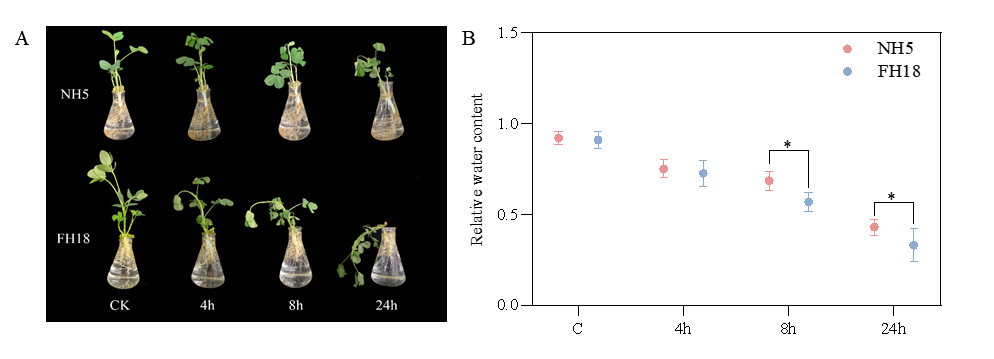


Fig.S3 Changes in the phenotype of peanut plants and the relative water content of leaves under drought stress
