# Supplementary material for: Differential photosynthetic responses to drought stress in peanut varieties: insights from transcriptome profiling and JIP-Test analysis
Source: BMC Plant Biol. 2025 Jul 25;25:957. doi: 10.1186/s12870-025-06984-y (PMC12291314; doi:10.1186/s12870-025-06984-y)
Supplement: Supplementary file 2 — Additional file 2: Table S1. The normalization of chlorophyll a fluorescence transient and relative parameters specific fluxes per active PSII reaction center. Table S2. GO annotation of differentially expressed genes in FH18. Table S3. GO annotation of differentially expressed genes in NH5. Table S4 Differentially expressed genes involved in photosynthesis in peanut. [file 12870_2025_6984_MOESM2_ESM.docx]

**Differential Photosynthetic Responses to Drought Stress in Peanut Varieties: Insights from Transcriptome Profiling and JIP-Test Analysis**

Jingyao Ren^1,2^, Pei Guo^2^, Xin Ai^2^, Xinlei Ma^2^, Jing Wang^2^, Xinhua Zhao^2^, Hongtao Zou^1^*, Haiqiu Yu^2,3^*

1. College of Land and Environment, Shenyang Agricultural University, Shenyang, China

2. College of Agronomy, Shenyang Agricultural University.Shenyang, China

3. Liaoning Agriculture Vocational and Technical College, Yingkou, China

*Corresponding author:

E-mail: hongtaozou208@163.com (H. Zou), [yuhaiqiu@syau.edu.cn](mailto:yuhaiqiu@syau.edu.cn) (H. Yu)

Table S1 The normalization of chlorophyll a fluorescence transient and relative parameters specific fluxes per active PSII reaction center

| Fluorescence parameter | Illustrations |
| --- | --- |
| V_OP_ | Induction curves double normalized of relative chlorophyll fluorescence (O-P) |
| V_OK_ | Induction curves double normalized of relative chlorophyll fluorescence (O-K) |
| W_OK_ | Reflect the L peak |
| V_OJ_ | Induction curves double normalised of relative chlorophyll fluorescence (O-J) |
| W_OJ_ | Reflect the K peak |
| V_t_ | Relative variable fluorescence at t point |
| ABS/RC | Light absorption flux per reaction centre (RC) |
| TRO/RC | Trapped energy flux per RC |
| ET_O_/RC | Maximum electron transport flux per PSII RC |
| RE_O_/RC | Electron flux reducing end electron accepters at PSI acceptor side per RC |
| DI_O_/RC | Dissipation energy flux per PSII RC |
| TRo/CSm | Trapped flux per CS |
| ETo/CSm | Electron transport flux per CS |
| REo/CSm | Electron flux reducing end electron acceptors at the PSI acceptor side per CS |
| DIo/CSm | Thermal dissipation energy flux per CS |
| φRo | Quantum efficiency of PSI-acceptor side end electron acceptor reduction |
| δRo | The efficiency of a single electron from the electron transport chain between optical systems to the end electron acceptor on the PSI acceptor side |
| φEo | Quantum efficiency of electron transport from QA to electron acceptor of electron transport chain except QA at t=0 |
| ψEo | The efficiency with which a single exciton captured by an active reaction center drives electron transport except for QA at=0 |
| PI_abs_ | Performance index (potential) for energy conservation from exciton to the reduction of intersystem electron acceptors |
| PI_tot_ | Performance index (potential) for energy conservation from exciton to the reduction of PSI end acceptors |

Table S2 GO annotation of differentially expressed genes in FH18

| GO_ID | GO_Term | GO_Category |  |
| --- | --- | --- | --- |
| GO:0006098 | pentose-phosphate shunt | Biological Process |  |
| GO:0019288 | isopentenyl diphosphate biosynthetic process, methylerythritol 4-phosphate pathway | Biological Process |  |
| GO:0016117 | carotenoid biosynthetic process | Biological Process |  |
| GO:0019344 | cysteine biosynthetic process | Biological Process |  |
| GO:0015995 | chlorophyll biosynthetic process | Biological Process |  |
| GO:0010207 | photosystem II assembly | Biological Process |  |
| GO:0009637 | response to blue light | Biological Process |  |
| GO:0010027 | thylakoid membrane organization | Biological Process |  |
| GO:0043085 | positive regulation of catalytic activity | Biological Process |  |
| GO:0009941 | chloroplast envelope | Cellular Component |  |
| GO:0009570 | chloroplast stroma | Cellular Component |  |
| GO:0019898 | extrinsic component of membrane | Cellular Component |  |
| GO:0009654 | photosystem II oxygen evolving complex | Cellular Component |  |
| GO:0009543 | chloroplast thylakoid lumen | Cellular Component |  |
| GO:0009535 | chloroplast thylakoid membrane | Cellular Component |  |
| GO:0009534 | chloroplast thylakoid | Cellular Component |  |
| GO:0016021 | integral component of membrane | Cellular Component |  |
| GO:0010598 | NAD(P)H dehydrogenase complex (plastoquinone) | Cellular Component |  |
| GO:0046658 | anchored component of plasma membrane | Cellular Component |  |
| GO:0030170 | pyridoxal phosphate binding | Molecular Function |  |
| GO:0005215 | transporter activity | Molecular Function |  |
| GO:0016851 | magnesium chelatase activity | Molecular Function |  |
| GO:0016161 | beta-amylase activity | Molecular Function |  |
| GO:0016705 | oxidoreductase activity, acting on paired donors, with incorporation or reduction of molecular oxygen | Molecular Function |  |
| GO:0051536 | iron-sulfur cluster binding | Molecular Function |  |
| GO:0004497 | monooxygenase activity | Molecular Function |  |
| GO:0042132 | fructose 1,6-bisphosphate 1-phosphatase activity | Molecular Function |  |
| GO:0009055 | electron carrier activity | Molecular Function |  |

Table S3 GO annotation of differentially expressed genes in NH5

| GO_ID | GO_Term | GO_Category |  |
| --- | --- | --- | --- |
| GO:0055114 | oxidation-reduction process | Biological Process |  |
| GO:0009637 | response to blue light | Biological Process |  |
| GO:0010218 | response to far red light | Biological Process |  |
| GO:0010114 | response to red light | Biological Process |  |
| GO:0016117 | carotenoid biosynthetic process | Biological Process |  |
| GO:0010207 | photosystem II assembly | Biological Process |  |
| GO:0000023 | maltose metabolic process | Biological Process |  |
| GO:0015996 | chlorophyll catabolic process | Biological Process |  |
| GO:0009773 | photosynthetic electron transport in photosystem I | Biological Process |  |
| GO:0016021 | integral component of membrane | Cellular Component |  |
| GO:0009941 | chloroplast envelope | Cellular Component |  |
| GO:0009535 | chloroplast thylakoid membrane | Cellular Component |  |
| GO:0009543 | chloroplast thylakoid lumen | Cellular Component |  |
| GO:0009654 | photosystem II oxygen evolving complex | Cellular Component |  |
| GO:0019898 | extrinsic component of membrane | Cellular Component |  |
| GO:0009534 | chloroplast thylakoid | Cellular Component |  |
| GO:0009570 | chloroplast stroma | Cellular Component |  |
| GO:0010287 | plastoglobule | Cellular Component |  |
| GO:0009538 | photosystem I reaction center | Cellular Component |  |
| GO:0016705 | oxidoreductase activity | Molecular Function |  |
| GO:0005506 | iron ion binding | Molecular Function |  |
| GO:0004497 | monooxygenase activity | Molecular Function |  |
| GO:0009055 | electron carrier activity | Molecular Function |  |
| GO:0005215 | transporter activity | Molecular Function |  |
| GO:0020037 | heme binding | Molecular Function |  |
| GO:0030170 | pyridoxal phosphate binding | Molecular Function |  |
| GO:0050660 | flavin adenine dinucleotide binding | Molecular Function |  |
| GO:0009011 | starch synthase activity | Molecular Function |  |

Table S4 Deferentially expressed genes involved in photosynthesis in peanut

| gene_name | FH18-4h | FH18-8h | FH18-24h | NH5-4h | NH5-8h | NH5-24h | Description |
| --- | --- | --- | --- | --- | --- | --- | --- |
| arahy.L9FPHH | 0.02 | -1.35 | -2.40 | 0.02 | -0.81 | -1.24 | photosystem II 22 kDa protein |
| arahy.RVN5Z1 | -1.76 | -2.48 | -2.78 | -2.00 | -2.52 | -2.15 | photosystem II reaction center W protein |
| arahy.T46WBJ | -0.87 | -1.98 | -4.40 | 0.20 | -1.28 | -3.12 | PsbQ-like protein 1 |
| arahy.G1SUYP | -0.13 | -2.33 | -3.42 | 0.66 | -1.39 | -2.58 | oxygen-evolving enhancer protein |
| arahy.Y7HUGW | -0.74 | -1.72 | -3.00 | -0.13 | -1.07 | -1.85 | oxygen-evolving enhancer protein 1 |
| arahy.TVDX40 | -1.23 | -2.01 | -4.16 | -1.21 | -1.40 | -3.48 | PsbQ-like protein 1 |
| arahy.QP7RRL | 0.06 | -1.14 | -2.72 | -0.29 | -0.88 | -1.83 | photosystem II 22 kDa protein |
| arahy.I21UC1 | -0.96 | -2.70 | -6.50 | -0.18 | -2.37 | -4.94 | PsbQ-like protein 1 |
| arahy.1944SK | -0.15 | -1.38 | -2.71 | 0.07 | -1.15 | -1.99 | oxygen-evolving enhancer protein |
| arahy.IUT8LB | -2.51 | -5.43 | -6.74 | -3.35 | -6.30 | -5.81 | PsbQ-like protein 1 |
| arahy.I4CVDG | -2.53 | -5.65 | -6.55 | -2.37 | -5.73 | -4.92 | PsbQ-like protein 1 |
| arahy.FRV526 | -2.29 | -2.85 | -2.17 | -2.68 | -3.28 | -1.22 | photosystem II reaction center PSB28 protein |
| arahy.HCTL36 | -0.13 | -0.37 | -2.06 | 0.64 | 0.12 | -1.19 | photosystem II 10 kDa polypeptide |
| arahy.XEQF22 | -2.01 | -2.29 | -3.04 | -1.18 | -1.60 | -1.39 | psbP-like protein |
| arahy.WM01M4 | -0.80 | -2.64 | -4.33 | 0.12 | -1.33 | -3.32 | PsbQ-like protein 1 |
| arahy.49ZYT6 | -0.69 | -1.84 | -3.61 | -0.21 | -1.26 | -2.53 | oxygen-evolving enhancer protein 1 |
| arahy.PIZC5V | -2.82 | -5.55 | -6.63 | -2.66 | -4.93 | -5.91 | PsbQ-like protein 1 |
| arahy.RBYB13 | -0.43 | -1.30 | -2.24 | 0.29 | -0.63 | -1.24 | oxygen-evolving enhancer protein 1 |
| arahy_new_19703 | 0.00 | 0.00 | 3.50 | 0.00 | 0.00 | 0.69 | photosystemIIQ(b)proteinD1 |
| arahy.72UZ71 | -1.67 | -2.15 | -1.77 | -1.74 | -1.95 | -0.70 | photosystem II reaction center PSB28 |
| arahy.TCR54Y | -2.04 | -2.97 | -3.26 | 0.00 | 0.00 | 0.00 | putative oxygen-evolving enhancer protein 2-2 |
| arahy.09KNS5 | -0.55 | -1.59 | -2.97 | -0.04 | -1.06 | -1.90 | oxygen-evolving enhancer protein 1 |
| arahy.1B2UBR | -0.13 | -1.00 | -2.67 | 0.56 | -0.40 | -2.36 | oxygen-evolving enhancer protein 3-2 |
| arahy.30A6BG | -0.21 | -1.58 | -3.13 | 0.08 | -1.17 | -2.34 | oxygen-evolving enhancer protein 2 |
| arahy.QDSR7W | -0.04 | -0.61 | -2.95 | 0.79 | -0.13 | -2.05 | photosystem I subunit O |
| arahy.71HCYD | 0.49 | -0.40 | -1.99 | 0.72 | -0.20 | -1.09 | photosystem I reaction center subunit VI-2 |
| arahy.V5WWJ8 | -0.76 | -2.84 | -4.58 | -0.59 | -2.56 | -3.85 | photosystem I reaction center subunit N |
| arahy.DHZ2RQ | -0.26 | -1.07 | -2.26 | 0.07 | -0.63 | -1.42 | photosystem I reaction center subunit IV |
| arahy_new_11593 | 6.93 | 6.89 | 7.43 | 0.00 | 0.00 | 5.30 | photosystem I P700 apoprotein A2 |
| arahy.YBSA05 | 0.76 | -0.24 | -1.72 | 1.04 | -0.02 | -1.05 | photosystem I reaction center subunit VI-2 |
| arahy.TN6DUA | 1.08 | 0.39 | -3.14 | 1.54 | 0.69 | -3.36 | photosystem I reaction center subunit IV |
| arahy.PI6GK2 | 0.08 | -1.22 | -2.81 | 0.53 | -0.57 | -1.95 | photosystem I reaction center subunit IV B |
| arahy.HB1P9N | 0.41 | -0.65 | -1.65 | 0.63 | -0.34 | -0.84 | photosystem I reaction center subunit IV B |
| arahy.MF538D | 0.10 | -0.86 | -3.00 | 0.44 | -0.31 | -2.17 | photosystem I reaction center subunit XI |
| arahy.PX7M3V | 0.06 | -1.13 | -2.71 | 0.05 | -0.96 | -1.77 | photosystem I reaction center subunit III |
| arahy.QW0L8Q | 1.27 | 0.60 | -2.22 | 1.08 | 0.75 | -2.25 | photosystem I reaction center subunit IV |
| arahy.9042LP | -0.53 | -1.60 | -3.03 | 0.32 | -1.04 | -2.11 | photosystem I reaction center subunit psaK |
| arahy.18YQBF | 0.17 | -0.69 | -2.82 | 0.37 | -0.27 | -2.04 | photosystem I reaction center subunit XI |
| arahy.DJM1RY | -0.92 | -2.98 | -4.64 | -0.74 | -2.72 | -4.06 | photosystem I reaction center subunit N |
| arahy.MVQ7LV | -0.08 | -0.54 | -2.74 | 0.74 | -0.05 | -1.75 | photosystem I subunit O |
| arahy.ZS1M1K | -0.36 | -1.57 | -3.34 | -0.35 | -1.93 | -2.90 | photosystem I reaction center subunit psaK |
| arahy.110G34 | 0.26 | -0.59 | -1.56 | 0.16 | -0.76 | -1.15 | photosystem I reaction center subunit IV |
| arahy.VHAI7W | 0.31 | -0.67 | -2.19 | 0.09 | -0.71 | -1.77 | photosystem I reaction center subunit III |
| arahy.4PD0PP | 0.18 | -1.02 | -2.27 | 0.05 | -0.83 | -1.21 | photosystem I reaction center subunit V |
| arahy_new_19267 | -0.40 | -1.35 | -2.86 | -0.33 | -1.13 | -1.96 | photosystem I reaction center subunit II |
| arahy.V1P8P6 | -0.44 | -1.23 | -2.47 | 0.12 | -0.78 | -1.59 | cytochrome b6-f complex iron-sulfur subunit |
| arahy.NN0MZU | -0.02 | -0.88 | -2.01 | 0.51 | -0.26 | -1.25 | cytochrome b6-f complex iron-sulfur subunit |
| arahy.39S8LV | -1.02 | -2.30 | -3.04 | -0.57 | -1.69 | -2.43 | cytochrome b6-f complex iron-sulfur subunit |
| arahy.F0UT86 | -0.92 | -1.52 | -1.89 | -0.37 | -0.98 | -1.25 | cytochrome b6-f complex iron-sulfur subunit |
| arahy.X5CP5W | -2.66 | -3.57 | -4.83 | -1.61 | -3.04 | -3.63 | plastocyanin |
| arahy.9X74H2 | 0.06 | 0.35 | 1.36 | 0.02 | 0.09 | 1.51 | ferredoxin |
| arahy_new_2177 | -2.33 | -3.28 | -3.35 | -2.84 | -3.06 | -2.79 | uncharacterized protein LOC107488537 |
| arahy.CW3FX9 | -2.87 | -5.50 | -6.47 | -3.20 | -5.56 | -7.17 | ferredoxin |
| arahy_new_20234 | 0.17 | 0.49 | 1.29 | -0.34 | -0.01 | 1.19 | ferredoxin, root R-B2 |
| arahy.61CDGC | -0.11 | -0.61 | -1.13 | 0.03 | 0.29 | -0.20 | ferredoxin--NADP reductase |
| arahy.AR01JJ | -0.88 | -2.28 | -4.30 | -0.50 | -1.39 | -3.26 | ferredoxin--NADP reductase |
| arahy.5F6K96 | -0.23 | -0.11 | -1.23 | -0.04 | 0.35 | -0.26 | ferredoxin--NADP reductase |
| arahy.ZQ3F6U | -0.56 | -1.93 | -4.11 | -0.49 | -1.26 | -3.21 | ferredoxin--NADP reductase |
| arahy.X3ZR55 | -0.88 | -0.85 | -2.27 | -1.01 | -1.39 | -1.34 | cytochrome c6 |
| arahy.8FEK6M | -1.30 | -1.17 | -1.73 | -1.21 | -1.03 | -1.21 | cytochrome c6 |
| arahy_new_6210 | -1.09 | -0.36 | -1.42 | -1.36 | -1.45 | -2.98 | photosystem I reaction center subunit II |
| arahy.KSV0XM | -1.56 | -2.79 | -3.70 | -1.29 | -2.55 | -2.95 | ATP synthase gamma chain |
| arahy.F6CLKD | -1.16 | -2.33 | -3.26 | -1.26 | -2.34 | -2.42 | ATP synthase gamma chain |
